# Contrasting the Microbial Communities in Rhizosphere and Bulk Soils Across Different *Eucommia ulmoides* Planting Sites and Their Soil Chemical Driving Mechanisms

**DOI:** 10.3390/microorganisms14081853

**Published:** 2026-08-20

**Authors:** Panfeng Liu, Huaxiang Wang, Furong Lin, Hongyan Du, Liwei Xing, Kunhao Xie, Qingxin Du

**Affiliations:** 1 State Key Laboratory of Tree Genetics and Breeding, Key Laboratory of Forest-Derived Medicinal Resources, National Forestry and Grassland Administration, Research Institute of Non-Timber Forestry, Chinese Academy of Forestry, Zhengzhou 450003, China; pfengliu@caf.ac.cn (P.L.); dhy515@caf.ac.cn (H.D.); m18405448176@163.com (L.X.); xiekunhao@163.com (K.X.); 2 State Key Laboratory of Efficient Production of Forest Resources, Research Institute of Fast-Growing Trees, Chinese Academy of Forestry, Zhanjiang 524022, China; wanghuaxiang2025@outlook.com; 3 Research Institute of Forestry, Chinese Academy of Forestry, Beijing 100091, China; linfr@caf.ac.cn

**Keywords:** soil multifunctionality, *Eucommia ulmoides*, microbial community, interaction co-occurrence network

## Abstract

Soil multifunctionality (SMF) is a core indicator of plantation soil ecological function, and microbial diversity plays a vital role in sustaining it. However, cross-site rhizosphere and bulk SMF disparities and their microbial driving mechanisms remain unclear in *Eucommia ulmoides* plantations. Here, we collected rhizosphere and bulk soils from three typical sites (Mengzhou, MZ; Liangyuan, LY; and Yuanyang, YY). Soil chemical properties, extracellular enzymes, microbial alpha diversity, community composition and cross-kingdom network topology were measured. Correlation heatmaps, random forest, Redundancy analysis (RDA) and Partial least path modeling (PLS-PM) were adopted to quantify SMF predictors and regulatory pathways. Rhizosphere soils possessed significantly higher alkaline hydrolyzable nitrogen (AN), available phosphorus (AP) and available potassium (AK) than bulk soils at all sites. LY rhizosphere showed the greatest soil organic carbon (SOC), total potassium (TK), available nutrients and enzyme activities, while YY had higher total nitrogen (TN) and AP, yet the lowest enzyme levels. Rhizosphere bacterial and fungal alpha diversity was consistently higher across locations. SMF varied distinctly by site and compartment: LY had substantially higher SMF than MZ and YY in both rhizosphere and bulk soils, with rhizosphere SMF being consistently greater than bulk values across all sites. The PLS-PM (GOF = 0.70) indicated that soil chemical properties regulated SMF via dual pathways: they directly promoted microbial co-occurrence networks and indirectly modified network structure by altering fungal diversity, while suppressing bacterial diversity. Total effect analysis identified soil chemical properties and microbial co-occurrence networks as the core drivers of SMF variation. This work clarifies that rhizosphere effects and soil chemical properties jointly drive SMF by regulating microbial diversity and co-occurrence networks, offering theoretical guidance for sustainable soil management in *E. ulmoides* plantations.

## 1. Introduction

Soil multifunctionality (SMF) refers to the integrated capacity of soil ecosystems to simultaneously supply, maintain and regulate multiple ecological services, primarily encompassing core ecological processes such as soil microbial activity, biogeochemical cycles of carbon and nitrogen, soil nutrient storage, nitrogen transformation, and litter decomposition [1]. As a comprehensive core indicator characterizing the health status and structural integrity of soil ecosystems, SMF is an essential component of terrestrial ecosystem multifunctionality. Compared with single soil function indicators, SMF can effectively integrate multidimensional environmental and ecological process information, accurately quantify the synergistic driving effects of various biological and environmental factors on soil ecological functions, and reveal the functional mechanisms of terrestrial ecosystems in a more comprehensive and systematic manner, thereby providing important theoretical support for the assessment of soil ecological value, ecosystem conservation, and refined management of planted forests [2].

Soil microbial communities are the key biological component driving core soil ecological processes, and they can mediate the formation, maintenance and dynamic evolution of SMF by regulating soil nutrient turnover, organic matter decomposition and aboveground primary production [3]. Currently, there is no unified consensus regarding the relationship between microbial diversity and SMF, and the relationship exhibits significant variations across habitat types and geographical variations. A global terrestrial ecosystem study confirmed that an increase in microbial diversity can enhance functional complementarity, optimize resource utilization efficiency, and significantly promote SMF [4]. However, other studies have found that excessively high microbial diversity intensifies interspecific niche competition and depletes limited soil resources, thereby inhibiting SMF [5]. With the development of microbial sequencing technology, the analysis of microbial co-occurrence networks has become a key approach for elucidating the relationship between microbial community structure and function, providing a new perspective for revealing the microbial regulatory mechanism of SMF [6]. Compared with traditional single diversity indices, topological characteristics of microbial networks, such as connectivity, complexity and modularity, can more accurately reflect microbial interspecific interaction patterns, community functional integration levels and ecological collaboration potential. Studies have demonstrated that complex microbial networks generally possess an efficient ecological functional coordination capacity and exhibit a significant positive correlation with SMF [7]. In addition, the stability (robustness) of both bacterial and fungal co-occurrence networks emerged as a critical biotic factor that was positively correlated with SMF of ancient *Juglans regia* [8]. Soil fungal diversity and abiotic factors drove SMF of *P. sylvestris* plantations, but abiotic factors were more important than biotic factors [2]. As a hotspot of microbial activity and nutrient turnover, the rhizosphere may reshape not only microbial community composition but also co-occurrence network topology, thereby providing a potential mechanistic link between root-mediated soil changes and ecosystem multifunctionality. Nevertheless, how rhizosphere effects specifically alter microbial network structure to drive SMF remains poorly understood.

*Eucommia ulmoides* Oliver, native to central and southern China, is the only species of genus (*Eucommia*) in the Eucommiaceae family, with multiple utilization values in medicinal use, natural rubber production and woody oil cultivation [9,10]. *E. ulmoides* trees are highly adaptive, and they naturally grow in China in the range of 25°–35° N and 104°–119° E. After introduction and domestication, now they can be planted in 27 subtropical to temperate provinces (autonomous regions and municipalities directly under the central government) in China [11]. Long-term artificial cultivation *E. ulmoides* significantly alters the composition of root exudates, changes the patterns of root nutrient uptake, and reshapes rhizosphere microenvironments, leading to significant differentiation in physicochemical properties between rhizosphere and bulk soil, which in turn drives the differential succession of soil microbial community structure and interaction patterns [12]. The composition of the fungal community, the relative abundance of species and the dominant taxa varied in the rhizosphere soil of *E. ulmoides* from different regions [13]. Studies on core microbials and their function in rhizosphere soil and bark-associated microbiota of *E. ulmoides* revealed that bark microbiota are primarily derived from rhizosphere soil. A total of 690 shared genera exist between rhizosphere soil and bark. The metabolic functions of the core microbial communities include ammonia oxidation, nitrogen fixation and polyhydroxybutyrate storage, all of which are closely related to plant growth and metabolism [14]. Furthermore, there were significant differences in physicochemical indicators between the rhizosphere and bulk soils of *E. ulmoides*, such as pH, electrical conductivity, total nitrogen and nitrate nitrogen. The nutrient content and microbial abundance in the rhizosphere soil were significantly higher than that in the bulk soil, confirming the evident rhizosphere effect of *E. ulmoides* [15].

Henan Province is a core production region for *E. ulmoides* in China, with significant spatial heterogeneity in climatic conditions, soil substrates and nutrient status among different sites. However, the differentiation characteristics of rhizosphere and bulk SMF and their microbial-driven mechanisms of *E. ulmoides* plantations across different regions still remain unclear. Accordingly, we selected three typical *E. ulmoides* plantations in Mengzhou City, Liangyuan District and Yuanyang County of Henan Province, and systematically determined soil chemical properties, soil enzyme activities, microbial diversity and community functional characteristics in rhizosphere and bulk soils across different regions. This study aims to reveal the spatial differentiation patterns of SMF in *E. ulmoides* plantations across different geographical habitats and elucidate the core mechanisms by which microbial communities and cross-kingdom networks regulate SMF. The research findings can provide a scientific basis and technical support for the regionalized intensive cultivation, soil fertility maintenance, ecological function improvement and quality optimization of *E. ulmoides* plantations. Meanwhile, it will enrich the theoretical framework of soil microbial ecology and multfunctionality regulation in economic forests.

## 2. Materials and Methods

### 2.1. Study Site Description and Sampling Collection

Three *Eucommia ulmoides* orchards in Mengzhou City (MZ), Liangyuan District (LY) and Yuanyang County (YY) of Henan Province were selected as experimental sites, where 8-year-old *E. ulmoides* cultivar ‘Huazhong 16’ was planted at a spacing of 4 m × 4 m. Environmental factors of the three sites are shown in Table 1. The plantations shared consistent cultivation practices after planting, including identical planting density, fertilization regimes, irrigation schedules, and pest management protocols, thereby minimizing management-related variability among sites. In August 2025, three 10 m × 10 m quadrats were set up in each experimental site. Five uniformly growing and healthy *E. ulmoides* trees were randomly selected in each quadrat. Surface litter was removed before sampling, and bulk soil samples were collected using the S-shaped sampling method. All sampling points were located 2 m away from the tree trunks, and soil samples were taken from the 0–20 cm soil layer using a soil auger. Rhizosphere soil was collected by rapidly shaking off the adhered soil from fine roots (with a diameter ≤ 2 mm). Then, five rhizosphere and bulk soils from each quadrat were fully homogenized and mixed into one composite sample, respectively. In total, three independent biological replicates were obtained for rhizosphere and bulk soil at each experimental site, respectively. All soil samples were preserved on dry ice and transported back to a laboratory. Each soil sample was divided into three portions: the first portion was stored at −80 °C for DNA extraction and high-throughput sequencing; the second portion was kept at 4 °C for soil enzyme activity assays; and the third portion was air-dried for the determination of soil physicochemical properties.

### 2.2. Soil Properties and Enzymatic Activity Analysis

Soil pH was determined in a 1:2.5 (*w*/*v*) soil–deionized water mixture using a pre-calibrated pH meter (Sartorius, Göttingen, Germany). The concentration of soil organic carbon (SOC) was analyzed through the wet oxidation colorimetric technique with a dichromate–sulfuric acid system [16]. Total nitrogen (TN) in soil samples was quantified after digestion and distillation based on the classic Kjeldahl protocol [17]. For total phosphorus (TP) detection, samples were subjected to sodium hydroxide fusion pretreatment prior to molybdenum–antimony colorimetric analysis [18]. Total potassium (TK) content was determined by flame photometry following alkaline fusion digestion [19].

For soil available nutrient determination, alkaline hydrolyzable nitrogen (AN) was measured using the alkaline diffusion method. Available phosphorus (AP) was extracted using the Mehlich 3 solution and quantified via molybdenum–antimony colorimetric assay [20]. Soil available potassium (AK) was extracted with ammonium acetate and subsequently quantified via flame spectrophotometry [19]. The activities of four functional soil enzymes involved in soil nutrient cycling, namely urease (URE), sucrase (SUC), catalase (CAT), and soluble neutral phosphatase (SNP), were determined using ultraviolet-visible spectrophotometry following standard soil biochemical protocols [21].

### 2.3. DNA Extraction and High-Throughput Sequencing

Total microbial genomic DNA was extracted from 0.25 g soil samples preserved at −80 °C using the E.Z.N.A.^®^ Soil DNA Isolation Kit (Omega Bio-tek, Norcross, GA, USA), and the purified DNA was resuspended in nuclease-free TE buffer. The concentration and purity of the obtained DNA were assessed using a NanoDrop ND 1000 spectrophotometer (NanoDrop Technologies, Wilmington, DE, USA). The bacterial 16S rRNA V3–V4 hypervariable region and fungal ITS1 region were amplified by polymerase chain reaction (PCR) with specific primer sets. Specifically, the bacterial amplification adopted the primers 338F (5′-ACTCCTACGGGAGGCAGCAG-3′) and 806R (5′-GGACTACHVGGGTWTCTAAT-3′), whereas the fungal ITS1 fragment was targeted using the primers ITS1 (5′-CTTGGTCATTAGAGGAAGTAA-3′) and ITS2 (5′-GCTGCGTTCTTCATCGATGC-3′). All PCR reactions were carried out on a T100 thermal cycler (Bio-Rad Laboratories, Hertfordshire, UK) following standardized thermal cycling protocols. Qualified PCR products were separated by 2% agarose gel electrophoresis, purified using the AxyPrep DNA Gel Extraction Kit (Axygen, Union City, CA, USA), and quantified with a Quantus Fluorometer (Promega, Madison, WI, USA). Equimolar purified amplicons were pooled and sequenced on the Illumina PE250 platform (Illumina, San Diego, CA, USA). Sequence processing was performed using QIIME 2 (version 2025.4). Raw paired-end reads were demultiplexed, and primer sequences were removed using the cutadapt plugin with a maximum error rate of 0.1 and a minimum overlap of 3 bp. Paired-end reads were subsequently denoised with the DADA2 plugin, wherein reads were truncated at 240 bp (forward) and 200 bp (reverse) and filtered at a maximum expected error (maxEE) of 2, and chimeric sequences were removed using the consensus method. Amplicon sequence variants (ASVs) were inferred directly from the denoised reads. Following ASV inference, singleton ASVs (represented by a single read across the entire dataset) and low-abundance ASVs (relative abundance < 0.005%) were removed to minimize spurious sequences. Taxonomic classification of ASVs was implemented via the RDP Classifier [22], with bacteria annotated against the Greengenes database and fungi against the UNITE database [23,24]. Good’s coverage was calculated to assess sequencing completeness, with values exceeding 0.99 across all samples indicating adequate sequencing depth. To eliminate quantitative bias, bacterial and fungal datasets were separately rarefied to their respective minimum sequencing depths: bacteria were rarefied to 24,760 sequences per sample, and fungi were rarefied to 38,935 sequences per sample.

### 2.4. Soil Multifunctionality

Soil multifunctionality (SMF) was quantified via the averaging method in this study. Twelve soil functional indicators closely relevant to soil nutrient provision and biogeochemical cycles were selected for SMF evaluation, including pH, SOC, TN, TP, TK, AN, AP, AK, URE, SUC, CAT, and SNP. For the calculation of average-based multifunctionality, Z-score standardization was performed on all twelve functional indices measured from soil samples. Briefly, the Z-score for each parameter was calculated as the difference between each individual measurement and the overall mean of the corresponding indicator, divided by its standard deviation. Notably, the Z-score for pH was inverted (multiplied by −1) prior to averaging to align its functional direction with the other indicators, thereby ensuring that all metrics contributed positively to the composite SMF index. The integrated SMF value of each soil sample was then determined by averaging the normalized Z-scores of the twelve functional metrics according to this standard calculation protocol [25].

### 2.5. Statistical Analyses

Prior to conducting parametric tests, normality of residuals was assessed using the Shapiro–Wilk test, and homogeneity of variances was evaluated using Levene’s test for all measured variables (pH, SOC, TN, TP, TK, AN, AP, AK, URE, SUC, CAT, SNP, and SMF). All variables satisfied these assumptions (*p* > 0.05), and one-way ANOVA combined with Tukey’s HSD test was subsequently applied to identify significant differences across different *E. ulmoides* planting regions and between rhizosphere and bulk soils. For multiple pairwise comparisons, *p* values derived from Spearman correlation analyses were adjusted using the Benjamini–Hochberg false discovery rate (FDR) method to control the family-wise error rate. Principal coordinate analysis (PCoA) based on bray_curtis distance metrics was implemented via the “ape” and “vegan” packages to visualize similarities and disparities in bacterial and fungal community structures across groups. Permutational multivariate analysis of variance (PERMANOVA) was further adopted to quantify the statistical significance of planting zone and rhizosphere effect on microbial community differentiation [26]. To improve the stability and statistical reliability of model estimation with a limited sample size, bootstrap resampling with 5000 iterations was performed. The standard errors and 95% bias-corrected confidence intervals of all path coefficients were calculated, and the statistical significance of each path was determined based on bootstrap testing. Microbial co-occurrence networks were constructed using the SparCC algorithm implemented in the SpiecEasi package in R (version 4.5.3), and network visualization was completed using the Gephi 9.3.1 software [27,28]. Redundancy analysis (RDA) was carried out using the R “vegan” package to elucidate the effects of soil chemical properties and enzyme activities on microbial community composition. The “randomForest” package along with Spearman correlation analysis was adopted to quantify the predictive contributions of soil chemical properties, enzyme activities, microbial diversity and network topological attributes to SMF [29]. The Random Forest regression model was implemented using the default configuration for regression tasks (ntree = 500, mtry = p/3, and minimum node size = 5). Variable importance was assessed via the permutation-based mean decrease in accuracy (%IncMSE). Model performance was evaluated using the out-of-bag (OOB) error estimate, yielding an OOB R^2^ of 0.35, supporting the reliability of the reported importance values. To reduce spurious correlations and improve network robustness, rare taxa (relative abundance < 0.01% or detected in fewer than three samples) were removed prior to analysis. Rarefaction normalization was conducted to unify sequencing depth prior to network construction. Co-occurrence networks were inferred using SparCC with 1000 bootstrap iterations to assess correlation significance; edges with |r| > 0.6 and *p* < 0.05 were retained to ensure robust associations. Topological differences among networks were presented descriptively, as each treatment yielded a single aggregated network. Partial least path modeling (PLS-PM) was executed through the “plspm” package to systematically disentangle the cascading pathways linking planting zones, rhizosphere microenvironments, soil functional traits, microbial community characteristics and SMF [30]. Multiple alternative path configurations were evaluated, and the final model was selected based on the highest goodness-of-fit (GoF) index. Model estimation used the path-weighting scheme with 500 bootstrap resamples to obtain 95% confidence intervals for path coefficients. The GoF index was calculated to assess the overall predictive reliability of the finalized PLS-PM [31].

## 3. Results

### 3.1. Rhizosphere and Bulk Soil Chemical Properties and Enzyme Activities of E. ulmoides at Different Sites

**Figure 1:**
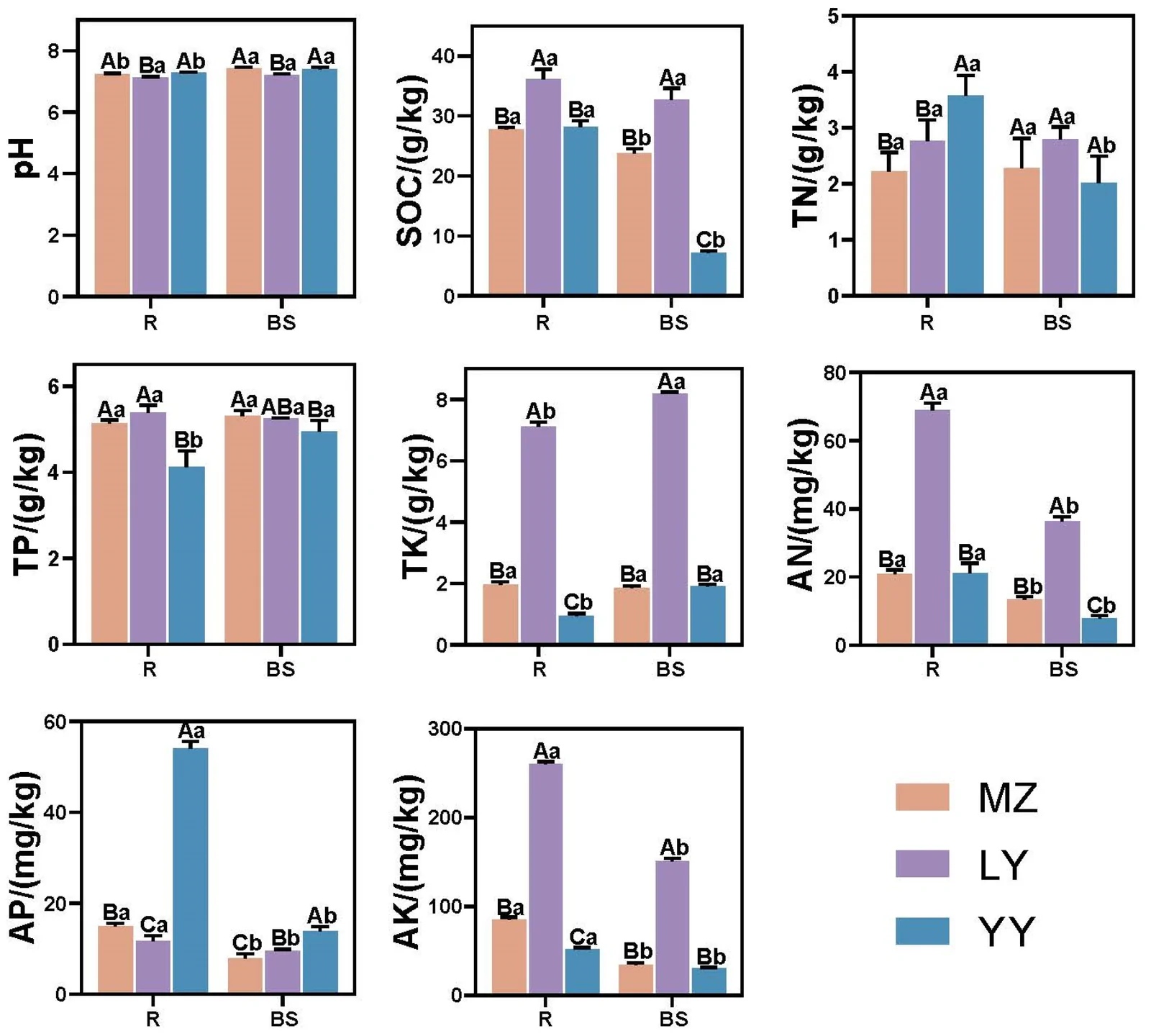
Soil chemical properties in rhizosphere (R) and bulk soils (BS) of *Eucommia ulmoides* at three sampling sites (MZ, LY, and YY). Different lowercase letters indicate significant differences between rhizosphere and bulk compartments (*p* < 0.05). Different uppercase letters indicate significant differences among the three sampling sites within the same soil compartment (*p* < 0.05). pH: soil pH value; SOC: soil organic carbon; TN: total nitrogen; TP: total phosphorus; TK: total potassium; AN: available nitrogen; AP: available phosphorus; AK: available potassium. MZ: Mengzhou; LY: Liangyuan; YY: Yuanyang.

Soil chemical properties of *E. ulmoides* were significantly influenced by both sampling location (MZ, LY, and YY) and soil compartment (rhizosphere vs. bulk) (*p* < 0.05; Figure 1). Regarding pH, values were significantly higher at MZ and YY than at LY across both compartments. Furthermore, bulk soils consistently exhibited a higher pH than rhizosphere soils at each site, with all values falling within a neutral to weakly alkaline range. Rhizosphere soils accumulate markedly higher available nitrogen (AN), available phosphorus (AP) and available potassium (AK) concentrations than bulk soils across all sites. Across both rhizosphere and bulk soils, the LY site contains significantly higher concentrations of SOC, total potassium (TK), AN, and AK relative to the MZ and YY sites. Across both rhizosphere and bulk soils, the LY site maintained significantly higher urease (URE), sucrase (SUC), catalase (CAT), and soluble neutral phosphatase (SNP) activities relative to the MZ and YY sites, consistent with the observed patterns in soil chemical properties (*p* < 0.05, Figure S1). The YY site generally exhibited the lowest enzyme activities, particularly in bulk soils, while MZ showed intermediate levels between LY and YY. Correlation analysis results (Figure S2) reveal that soil enzyme activities (URE, SUC, CAT, and SNP) exhibited significant negative correlations with soil pH, whereas they were significantly positively correlated with soil SOC, TK, AN, and AK (*p* < 0.05).

### 3.2. Soil Microbial Diversity and Community Composition

**Figure 2:**
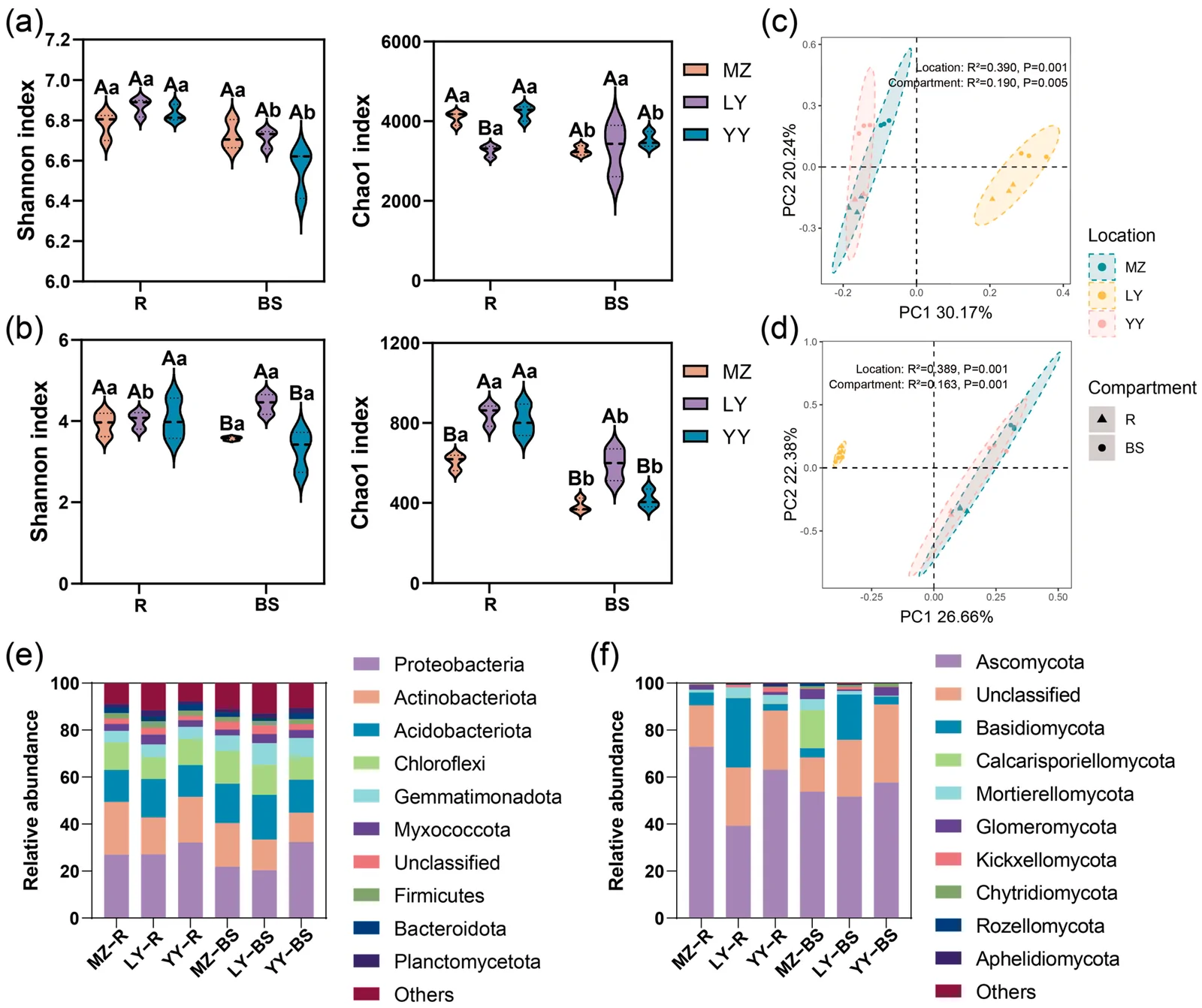
Soil microbial alpha diversity (**a**,**b**), beta diversity (**c**,**d**), and species composition (**e**,**f**) in response to sampling locations, rhizosphere and bulk soils. Different lowercase letters indicate significant differences between rhizosphere and bulk compartments (*p* < 0.05). Different uppercase letters indicate significant differences among the three sampling sites within the same soil compartment (*p* < 0.05). Ellipses represent the 95% confidence interval for each group. MZ: Mengzhou; LY: Liangyuan; YY: Yuanyang; R: rhizosphere soil; BS: bulk soil.

Alpha diversity (Shannon and Chao1 index) of soil bacterial and fungal communities differed significantly across sampling locations and between rhizosphere and bulk soils (*p* < 0.05, Figure 2a,b). Bacterial and fungal alpha diversity were markedly higher in rhizosphere soils than bulk soils at the majority of sampling locations. The bacterial Chao1 index in rhizosphere compartment was significantly lower at the LY site relative to the MZ and YY sites, while no obvious inter-site difference was detected in bulk soils. For fungi, both Shannon and Chao1 indices in bulk soils were significantly higher at the LY site compared with MZ and YY. Additionally, MZ and LY harbored a substantially greater fungal Chao1 index than YY within rhizosphere soils. Principal coordinate analysis (PCoA) coupled with PERMANOVA indicated that bacterial and fungal community compositions were distinctly shaped by sampling location and rhizosphere compartment. Sampling location was the stronger driver for both bacteria (R^2^ = 0.390, *p* = 0.001) and fungi (R^2^ = 0.369, *p* = 0.001), while rhizosphere compartment also induced significant community differentiation for bacteria (R^2^ = 0.190, *p* = 0.005) and fungi (R^2^ = 0.163, *p* = 0.001) (Figure 2c,d). The dominant bacterial phyla across all soil samples included Proteobacteria, Actinobacteriota, Acidobacteriota, Chloroflexi, and Gemmatimonadota, whereas the fungal community was predominated by Ascomycota, Basidiomycota, Calcarisporiellomycota, and Mortierellomycota (Figure 2e,f). Significant variations in the relative abundances of dominant fungal phyla were observed across sampling locations and between rhizosphere and bulk soils (Figures S3 and S4, *p* < 0.05). For bacteria, the LY site exhibited relatively lower relative abundances of Proteobacteria, Actinobacteriota, and Bacteroidota, while harboring higher relative abundances of Gemmatimonadota and Myxococcota compared with the MZ and YY sites. For fungi, rhizosphere communities at LY were distinguished by significantly lower Ascomycota but higher Basidiomycota abundances, while YY soils accumulated more Kickxellomycota and Aphelidiomycota. In bulk soils, LY exhibited reduced Glomeromycota but elevated Kickxellomycota, and MZ harbored higher abundances of Calcarisporiellomycota, Mortierellomycota, and Rozellomycota.

### 3.3. Soil Microbial Co-Occurrence Network

**Figure 3:**
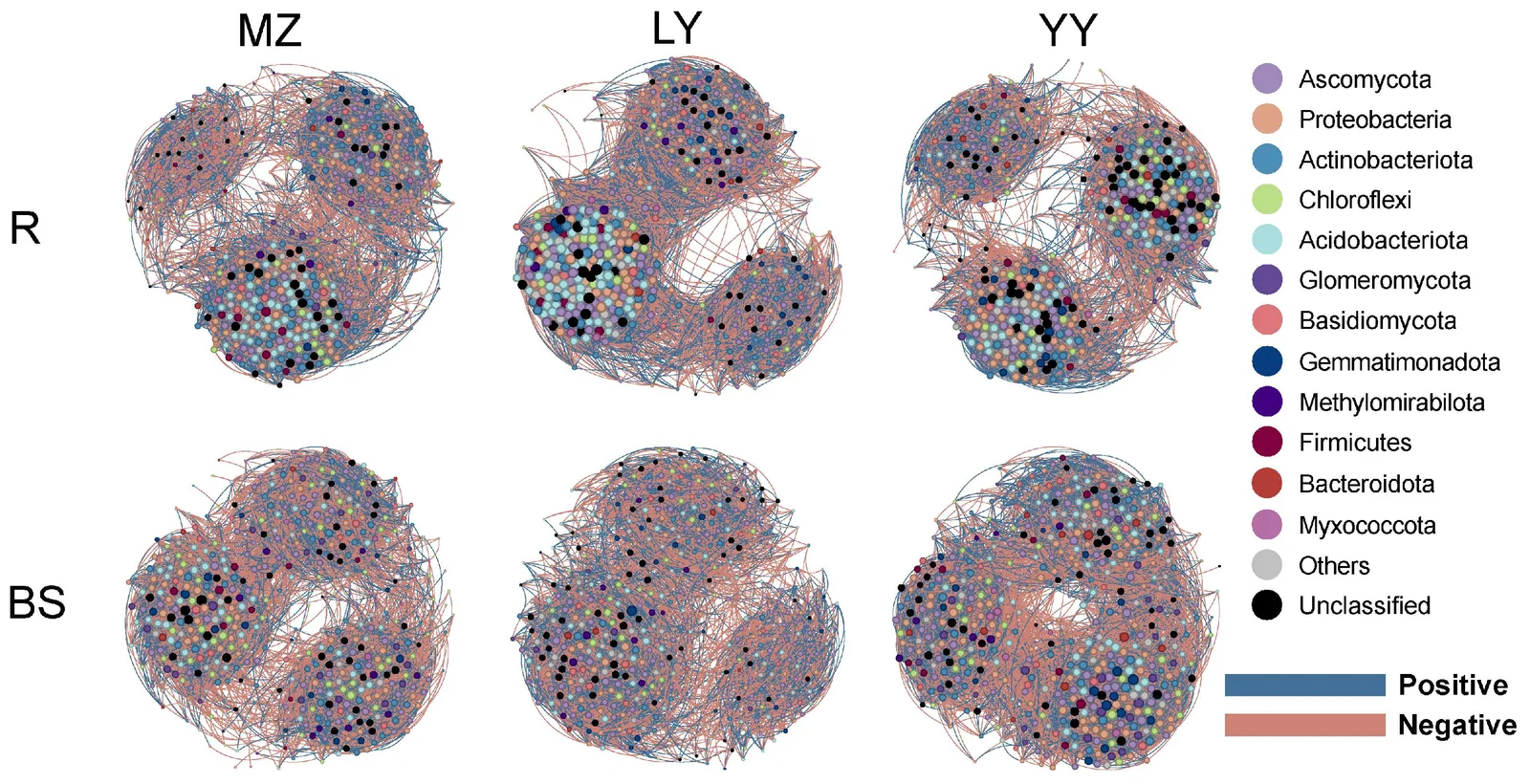
Soil bacterial–fungal co-occurrence networks across *E. ulmoides* planting locations and two soil compartments, with nodes colored by microbial phylum and edges representing positive (blue) and negative (red) correlations. R: rhizosphere soil; BS: bulk soil. MZ: Mengzhou; LY: Liangyuan; YY: Yuanyang.

Bacterial and fungal cross-kingdom co-occurrence networks were established across sampling locations and between rhizosphere and bulk soils (Figure 3). Bacterial nodes were primarily composed of Proteobacteria, Actinobacteriota, Chloroflexi and Acidobacteriota, and fungal nodes were dominated by Ascomycota, Basidiomycota and Glomeromycota. Topological analysis (Figure S5) showed that in rhizosphere soil, LY had the highest Edge, Connectance, Average degree, Centralization degree, and Centralization closeness. In bulk soil, LY presented the lowest Edge, Connectance, Average degree, Average clustering coefficient and Relative modularity but possessed a relatively higher Average path length, Centralization degree, Centralization betweenness and Centralization closeness relative to MZ and YY. By contrast, MZ and YY shared similar topological characteristics for most network parameters in bulk soil.

### 3.4. Relations Between the Microbial Community Structure and Soil Properties

**Figure 4:**
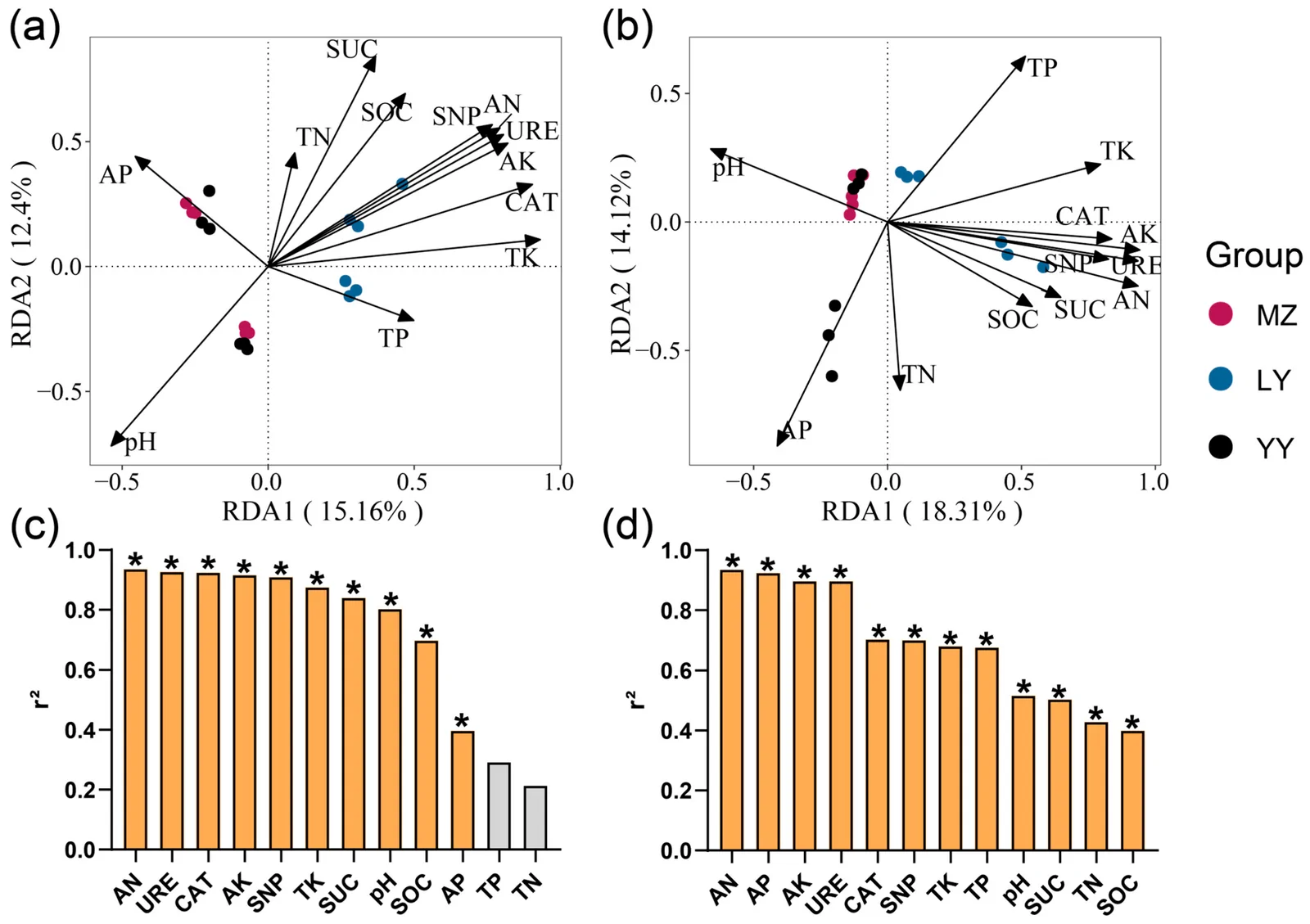
Redundancy analysis (RDA) and key soil properties of bacterial and fungal communities. (**a**,**b**) RDA ordination plots of bacterial (**a**) and fungal (**b**) communities in relation to soil chemical properties and enzyme activities. Samples are colored by planting site. (**c**,**d**) Relative explanatory power (r^2^) of environmental variables for bacterial (**c**) and fungal (**d**) community structures, with asterisks indicating significant explanatory variables (*p* < 0.05).

Redundancy analysis (RDA) revealed distinct soil properties shaping bacterial and fungal community structures (Figure 4a,b). Variation partitioning analysis confirmed the relative importance of these factors (Figure 4c,d). For bacteria, AN, URE, CAT, AK, SNP, TK, SUC, pH, SOC, and AP were the most significant explanatory variables, whereas for fungi, AN, AP, AK, URE, CAT, SNP, TK, TP, pH, SUC, TN, and SOC exhibited the strongest explanatory power, highlighting the dominant role of soil nutrients and enzyme activities in structuring microbial communities.

Correlation heatmap analysis revealed the associations between soil chemical properties, enzyme activities, and bacterial alpha diversity as well as phylum-level community composition (Figure S6). Bacterial Shannon index, Myxococcota, fungal Shannon and chao1 index, and Basidiomycota were significantly positively correlated with SOC, AN, AK, URE, SUC, CAT and SNP and significantly negatively correlated with soil pH (*p* < 0.05). Bacterial PC2, fungal PC1 and Glomeromycota presented contrary correlation patterns. Furthermore, a correlation heatmap was further constructed to disentangle the associations between soil chemical properties, enzyme activities and all topological metrics of cross-kingdom networks (Figure S7). The results indicate that Centralization degree and Centralization closeness had significant negative correlations with soil pH, yet displayed prominent positive correlations with SOC, AN, AK, URE, SUC, CAT, and SNP. In addition, AP showed significant positive associations with Edge, Connectance, Average degree, and Relative modularity.

### 3.5. Direct and Indirect Effects of Soil Multifunctionality Drivers

**Figure 5:**
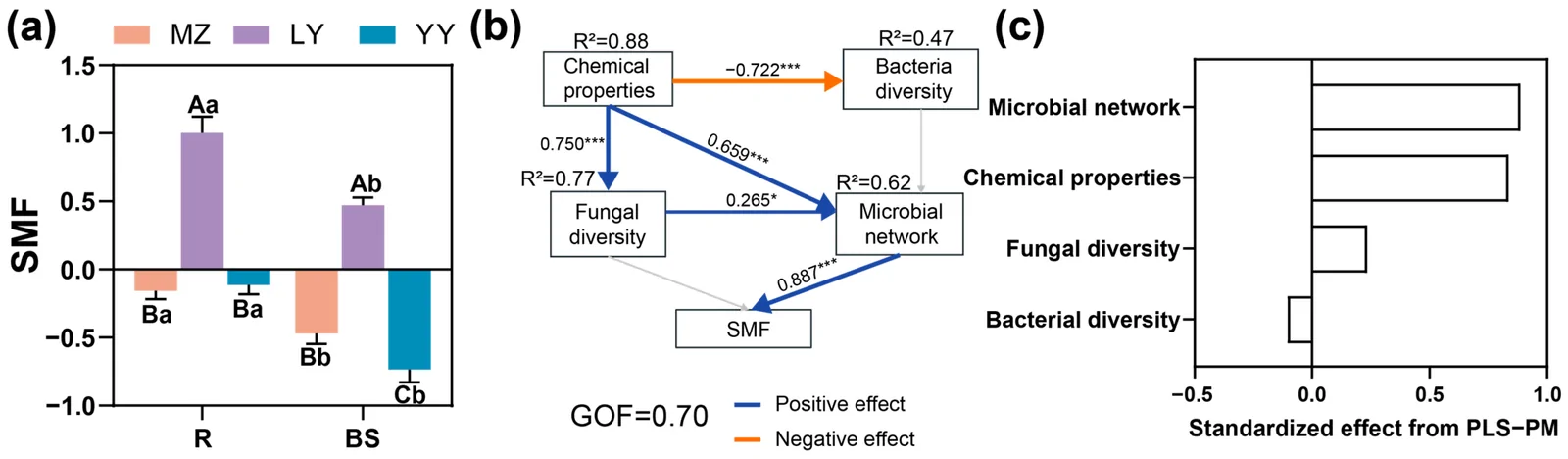
Soil multifunctionality (SMF) and its driving mechanisms across different sites. (**a**) Differences in SMF among three planting sites in rhizosphere and bulk soils. (**b**) Partial least squares path modeling (PLS-PM) reveals the direct and indirect impacts of soil chemical properties, microbial diversity, and microbial interaction networks on SMF. (**c**) Standardized total effects of all predictors on SMF derived from the PLS-PM analysis, quantifying the relative contribution of each factor to SMF variation. Uppercase letters indicate significant differences among planting sites within the same soil compartment, while lowercase letters indicate significant differences between rhizosphere and bulk soils within the same site (*p* < 0.05). Blue and orange arrows indicate positive and negative effects, respectively. The percentage near each module indicates the in-sample proportion of variance explained (R^2^). * *p* < 0.05, *** *p* < 0.001. MZ: Mengzhou; LY: Liangyuan; YY: Yuanyang.

Soil multifunctionality (SMF) exhibited distinct variations across planting locations and two soil compartments (Figure 5a). In rhizosphere soil, LY exhibited significantly higher SMF than MZ and YY (*p* < 0.05). In bulk soil, SMF followed the order LY > MZ > YY. Notably, all sampling sites possessed markedly higher SMF in rhizosphere relative to bulk soil. Correlation heatmap and random forest analysis were combined to clarify the associations between SMF and multiple variables, including soil chemical properties, enzymatic activities, microbial alpha diversity, microbial community composition, and topological characteristics of microbial co-occurrence networks (Figure S8).

The results demonstrate that soil properties including AN, TK, AK, SOC, URE, SNP, CAT and pH were significantly correlated with SMF, and random forest analysis further verified that these indicators acted as significant key predictors of SMF. Topological properties of microbial interaction networks also served as important predictors for SMF variation. With regard to microbial diversity and community composition, the bacterial Shannon index, Myxococcota, fungal Shannon, chao1 index, Basidiomycota, Glomeromycota, Kickxellomycota, and Aphelidiomycota all exhibited significant correlations with SMF (*p* < 0.05).

A partial least squares path model (PLS-PM) was established to disentangle the direct and indirect driving mechanisms underlying SMF (Figure 5b), and the goodness-of-fit (GOF = 0.70) validated that this model possessed strong interpretability. PLS-PM analysis demonstrated the multiple pathways by which soil chemical properties regulate SMF. Primarily, soil chemical properties displayed significant direct positive impacts on microbial co-occurrence networks. Moreover, soil chemical properties could indirectly reshape microbial network structure through mediating fungal diversity, and such shifts in microbial networks ultimately drove changes in SMF. Separately, soil chemical properties had a remarkable negative direct correlation with bacterial diversity. Total effect analysis showed that microbial co-occurrence networks and soil chemical properties were the main drivers of SMF variation (Figure 5c).

## 4. Discussion

### 4.1. Rhizosphere Effect and Site Specificity Modulate Soil Nutrients and Enzyme Activities in E. ulmoides Plantations

The obvious differences in soil nutrients and enzyme activities between rhizosphere and bulk soils are mainly driven by rhizosphere microhabitat changes induced by plant root activities (Figure 1). Plant roots in rhizosphere zones continuously secrete root secretions, such as organic acids, sugars and amino acids, from *E. ulmoides*. These substances increase organic carbon substrates in rhizosphere soils [32]. Meanwhile, selective uptake of mineral nutrients by roots alters ion balance and microenvironmental conditions around roots, leading to nutrient accumulation in rhizosphere zones [33]. In this study, rhizosphere soils at all three sites contained significantly higher SOC, AN and AK concentrations than bulk soils, accompanied by much higher activities of urease, sucrase, catalase and neutral phosphatase (Figure S1). The consistent variation of nutrients and enzyme activities indicates that soil nutrient pools and extracellular enzymes do not function independently but interact as an integrated cycling system.

Extracellular enzymes secreted by soil microorganisms decompose organic substrates and mineralize nitrogen, phosphorus and potassium. Abundant organic carbon and available nutrients act as substrates to stimulate microorganisms to produce enzymes related to carbon, nitrogen and phosphorus cycling [34]. In turn, increased enzyme activities accelerate the decomposition of soil organic matter and promote the release of available nutrients, thereby establishing a positive feedback relationship. Nutrient accumulation stimulates higher enzyme activity, which speeds up nutrient transformation and circulation and ultimately facilitates additional nutrient accumulation in soils [35]. Our correlation analysis supports this interactive relationship: URE, SUC, CAT and SNP all showed significant positive correlations with SOC, TK, AN and AK (Figure S2). This finding suggests that sufficient nutrients support high enzyme activity, while intensive enzymatic reactions speed up nutrient turnover and continuously replenish labile nutrients in rhizosphere soils. Our study detected significant negative correlations between all measured enzyme activities and soil pH (Figure S2), indicating that mild near-neutral soil conditions are favorable for sustaining high enzymatic activity, whereas strongly alkaline environments tend to suppress enzyme performance [36]. Consistent with previous research highlighting soil pH as a core regulator of soil biogeochemistry, pH governs microbial communities, extracellular enzyme activity and SOM stabilization dynamics, thereby acting as a decisive factor shaping soil nutrient transformation [37]. The higher SOC, TN, AN, and AK at LY reflect optimized enzyme activities under its comparatively lower soil pH, which accelerated organic matter decomposition and nutrient mineralization [38]. In contrast, AP peaked in the YY rhizosphere because P availability is governed by mineral solubilization rather than organic matter decomposition; Rhizosphere acidification likely dissolved mineral-bound P under the low-nutrient conditions at this site.

### 4.2. Spatial Variation and Rhizosphere Effects on Soil Microbial Communities

Soil microbial communities in the *E. ulmoides* plantations exhibited pronounced spatial and rhizosphere-dependent differentiation, as evidenced by significant variations in alpha diversity, community composition, and co-occurrence network topology across sampling locations and soil compartments (Figure 2). Bacterial and fungal alpha diversity were consistently higher in rhizosphere soils than in bulk soils at most sampling locations, reflecting the well-documented rhizosphere effect driven by root exudate inputs. However, the magnitude of this effect varied markedly across sampling locations, suggesting that site-specific soil properties, including pH, texture, and mineral composition, act as fundamental environmental filters that constrain the baseline microbial community and modulate the expression of rhizosphere enrichment. The rhizosphere represents a unique microenvironment where plants actively deposit labile carbon compounds, including sugars, organic acids, and amino acids, which serve as readily available energy sources for microbial growth and activity [39]. This enhanced resource availability not only supports larger microbial populations but also fosters greater taxonomic richness by reducing competitive exclusion and enabling the coexistence of metabolically diverse taxa [40]. The higher Shannon and Chao1 indices in rhizosphere soils likely arise from the combined influence of labile carbon inputs from root exudates and the spatially variable availability of soil nutrients, particularly nitrogen and phosphorus, which create heterogeneous metabolic niches that support metabolically diverse taxa [41]. Furthermore, mycorrhizal fungi amplify the divergence between rhizosphere and bulk soils through the metabolic and physical architecture of their extraradical hyphal networks. Extending well beyond the root depletion zone, these hyphae establish a distinct hyphosphere microenvironment enriched with carbon substrates including glomalin-related soil proteins, hyphal exudates, and necromass, which differ in chemical quality and turnover dynamics from root-derived inputs [42]. This hyphal imprint reshapes microhabitat heterogeneity and substrate accessibility, thereby imposing divergent selective pressures that further magnify the differentiation between rhizosphere and bulk soils in both nutrient status and community structure. At the phylum level, Basidiomycota were notably more abundant in rhizosphere than in bulk soils. This enrichment likely reflects the selective proliferation of rhizosphere-adapted basidiomycetous taxa fueled by the continuous input of labile carbon from *E. ulmoides* root exudates, coupled with the enhanced nutrient availability that facilitates their establishment in the root microenvironment. Moreover, in forest ecosystems, this classical rhizosphere effect is substantially governed by root-associated mycorrhizal fungi. These fungi intercept root-derived photosynthates to sustain extensive extraradical hyphal networks [43]. Such networks expand the rhizosphere footprint and act as selective hotspots that recruit mycorrhiza-helper bacteria (MHB) through specialized metabolites and unique carbon niches [44]. This process effectively filters rhizosphere microbial assemblages [45]. Consequently, the observed divergence between rhizosphere and bulk soils likely reflects not merely enhanced substrate availability but divergent community assembly rules underpinned by mycorrhizal-mediated biotic filtering and metabolic cross-feeding.

Microbial diversity and community composition displayed distinct spatial heterogeneity across the three *E. ulmoides* planting sites, with bacteria and fungi exhibiting divergent biogeographic distribution patterns. Consistent with the core tenet of global soil microbial biogeography, edaphic nutrient filtering serves as a fundamental driver of microbial community assembly and spatial differentiation across landscapes [46]. Variable soil nutrient status among sampling sites creates heterogeneous environmental niches, which exert strong selective pressure and reshape the taxonomic structure and diversity of bacterial and fungal communities [47]. Microbial taxa exhibit divergent nutrient adaptation strategies [48]: copiotrophic bacteria thrive in nutrient-rich soils but tend to decline in diversity under nutrient-limited conditions, while fungi with flexible trophic strategies and hyphal foraging capacities are more tolerant of resource scarcity, thereby sustaining or even elevating fungal diversity in low-nutrient soils [41,49]. Consistent with these ecological mechanisms, RDA analysis further verified that the spatial variation of soil bacterial and fungal communities was predominantly driven by multiple soil nutrient indicators (Figure 4), strongly supporting that differential nutrient availability across planting regions is the decisive environmental factor shaping microbial biogeographic patterns in *E. ulmoides* plantation soils.

### 4.3. Soil Properties and Microbial Network Topological Properties Co-Regulate Soil Multifunctionality via Cascading Pathways

PLS-PM results confirm that microbial co-occurrence networks and soil nutrient served as the core dominant drivers shaping SMF gradients and constructed a multi-step cascading regulatory pathway (Figure 5). This finding aligns well with the long-standing principle in soil ecology that community structure determines ecosystem function, though the specific mechanisms behind this relationship need to be unpacked at the network level [50]. Microbial co-occurrence networks capture how species associate with one another in nature: positive linkages may reflect cooperative processes such as cross-feeding, joint substrate degradation, and quorum sensing, whereas negative linkages signal competitive exclusion, antagonism, or niche partitioning [7]. Network topology integrates these interaction patterns into a single measurable index, effectively gauging how efficiently a community turns its latent functional capacity into actual output [51]. Meanwhile, more complex networks show stronger resistance to environmental disturbances; potentially accommodate diverse putative interaction forms consistent with mutualism, competition, parasitism and predation; and generate greater functional complementarity, all of which drive the coordinated expression of multiple ecological functions [52]. Positive correlations may plausibly represent potential cooperative relationships such as cross-feeding, whereas negative correlations could imply putative competition or antagonism. However, the strong negative direct effect of soil chemical properties on bacterial diversity indicates that high nutrient levels selectively promote fast-growing copiotrophic taxa while competitively excluding slow-growing oligotrophs, leading to community homogenization and suppressed bacterial richness. Conversely, simplified microbial networks may lead to functional homogenization among species, weaken the community’s capacity to cope with environmental risks, and ultimately reduce soil multifunctionality [53]. This general mechanism is particularly pronounced in the *E. ulmoides* planting system, where root inputs and rhizosphere processes strongly modulate microbial interactions. As a medicinal woody species, *E. ulmoides* produces abundant phenolics, terpenoids and polysaccharides, a suite of metabolites readily released into rhizosphere via root exudation. Its rhizosphere chemical signature differs from common broadleaf and coniferous trees, favouring cooperative microbial associations and elevated network connectivity, thereby strengthening the linkage between network stability and soil multifunctionality [14,54].

In this regulatory cascade, fungal diversity acts as a critical indirect mediator bridging soil physicochemical properties and microbial network topology (Figure 5b). Soil chemical variables function as key environmental filters that shape fungal community assembly: soil pH selects for acid- or alkali-tolerant fungal taxa, soil organic matter regulates the abundance of saprotrophic and mycorrhizal fungi, and nutrient stoichiometry constrains fungal metabolic strategies [55]. These chemical gradients drive structural variation in fungal communities, and the resulting shifts in fungal diversity further reorganize microbial co-occurrence networks by enhancing positive interspecific associations, strengthening module coupling, and improving phylogenetic functional redundancy [56]. The restructured microbial networks ultimately translate soil property and fungal diversity signals into soil multifunctionality via modular functional partitioning and keystone species regulation [57].

In the *E. ulmoides* planting system, soils from different geographical origins shape differentiated organizational patterns of microbial networks, and the evolution of these network structures exhibits a significant positive synergistic relationship with the dynamic changes of soil chemical properties (Figure 5b). Improved nutrient availability weakened resource competition, enabling taxa that would otherwise exclude one another under scarcity to coexist and form cooperative partnerships. The proportion of positive associations consequently rose, and the network architecture shifted from loose and modular toward tight and integrated [58]. Such densely connected networks were consistent with potential metabolite sharing, cross-feeding, and functional complementarity, which enhanced resource-use efficiency and accelerated material cycling, thereby maximizing soil multifunctionality under nutrient-rich conditions [59]. By contrast, nutrient-poor soils supported networks of lower connectivity. Although this sparser architecture constrained system-wide functional coordination, it effectively buffered local disturbances from propagating across the entire network, preserving a baseline level of soil multifunctionality under resource-limited stress [60]. In conclusion, these results demonstrate that soil nutrients indirectly govern the spatial patterning of soil multifunctionality by reshaping microbial interaction architectures. Notably, soil properties primarily regulate soil multifunctionality through such biological mediation pathways rather than through simple direct effects, with fungal diversity and remodeled microbial network topology serving as the pivotal intermediate links in the multi-step regulatory chain.

## 5. Conclusions

This study investigated the differences in soil chemical properties, extracellular enzyme activities, microbial communities, and soil multifunctionality (SMF) among three *E. ulmoides* plantation sites, as well as between rhizosphere and bulk soils, and further elucidated the key mechanisms driving SMF variation. Across all sampling sites, rhizosphere soils contained significantly higher available nutrients and harbored greater bacterial and fungal alpha diversity than bulk soils. Obvious site-dependent heterogeneity in soil nutrients, enzyme activities and SMF was detected; specifically, the Liangyuan site exhibited the greatest nutrient status, enzymatic activity and overall SMF. PLS-PM analysis revealed that soil chemical properties regulated SMF through two distinct pathways: they directly modulated microbial co-occurrence network structure and indirectly altered network topology by differentially regulating fungal and bacterial diversity. Overall, soil chemical properties and microbial co-occurrence networks served as the core drivers of SMF variation. Collectively, this study demonstrates that rhizosphere effects and soil chemical properties jointly drive SMF through their regulation of microbial diversity and co-occurrence networks, providing a theoretical basis for sustainable soil management in *E. ulmoides* plantations. For example, targeted organic amendments can be implemented at the Yuanyang site. Organic inputs are capable of enhancing soil biological activity, stimulating the production of extracellular enzymes, and alleviating nutrient limitations.

Nevertheless, this study selected only one plantation site per region at a single time point, and 18 samples does not meet the conventional threshold of 30–50 observations for stable PLS-PM path coefficient estimation, which may limit the generalizability of the identified soil–microbe regulatory relationships. In future work, we will investigate the seasonal dynamics and diverse stand ages of microbial communities and integrate functional metagenomics and metatranscriptomics to uncover the causal mechanisms linking soil properties, microbial interactions and soil multifunctionality.

## Figures and Tables

**Table 1:** Environmental factors of study sites.

Study Sites	Longitude/E	Latitude/N	Average Annual Temperature/°C	Mean Annual Precipitation/mm	Altitude/m	Soil Texture
MZ	112°42′58″	34°51′38″	14.2	586.9	100.5	sandy loam
LY	115°30′40″	34°34′18″	13.9	700	49	mixed loam
YY	113°47′35″	34°55′27″	14.4	549.9	78	sandy loam

## Data Availability

The sequencing data presented in this study are openly available in NCBI under BioProject accession PRJNA1510896 and PRJNA1510919.

## Supplementary Materials

The following supporting information can be downloaded at: https://www.mdpi.com/article/10.3390/microorganisms14081853/s1, Figure S1: Soil enzyme activities in rhizosphere (R) and bulk soils (BS) of *Eucommia ulmoides* at three sampling sites (MZ, LY, YY); Figure S2: Heatmap of correlations of soil properties with enzyme activities; Figure S3: Relative abundance of bacterial phyla with significant variation across sampling sites and rhizosphere compartments; Figure S4: Relative abundance of fungal phyla with significant variation across sampling sites and two soil compartments; Figure S5: Microbial co-occurrence network topological properties differ between sampling sites and rhizosphere compartments; Figure S6: Heatmap of correlations of soil properties, enzyme activities with bacterial/fungal diversity and community composition; Figure S7: Heatmap of correlations of soil properties and enzyme activities with microbial co-occurrence network topological properties; Figure S8: Correlation and random forest analysis of factors associated with soil multifunctionality.
