# Supplementary material for: Contrasting the Microbial Communities in Rhizosphere and Bulk Soils Across Different Eucommia ulmoides Planting Sites and Their Soil Chemical Driving Mechanisms
Source: Microorganisms. 2026 Aug 20;14(8):1853. doi: 10.3390/microorganisms14081853 (PMC13515387; doi:10.3390/microorganisms14081853)
Supplement: Supplementary file 1 [file microorganisms-14-01853-s001.zip › microorganisms-4472269-supplementary.pdf]

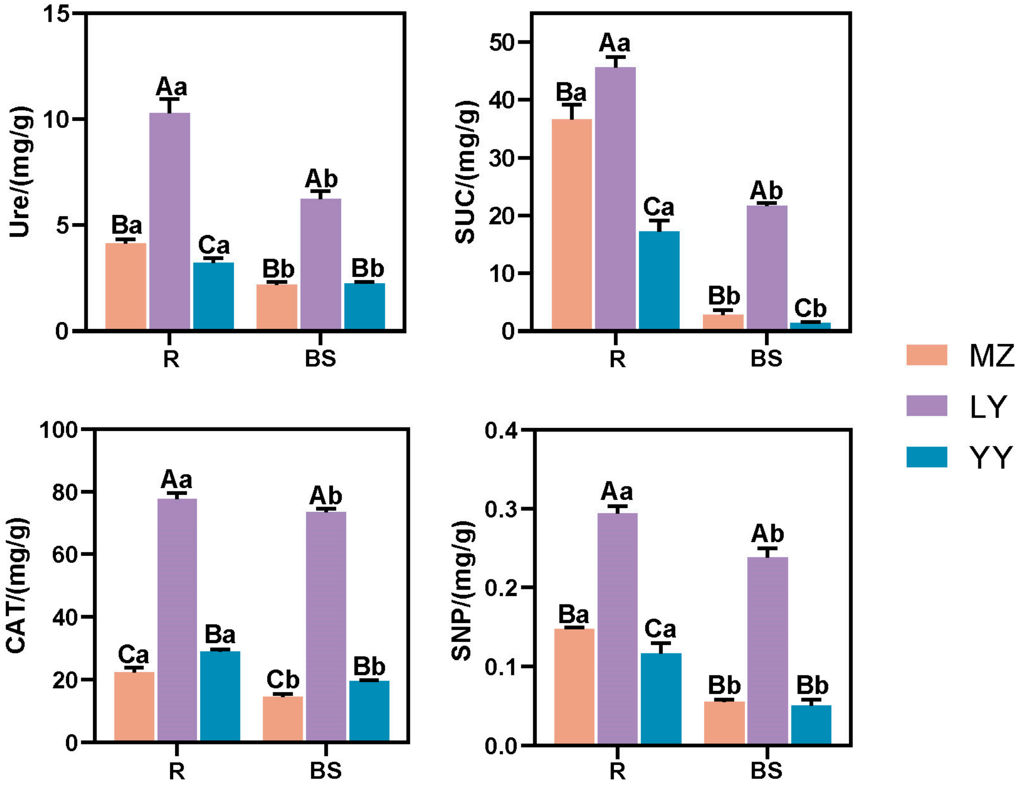

Figure S1. Soil enzyme activities in rhizosphere (R) and bulk soils (BS) of *Eucommia ulmoides* at three sampling sites (MZ, LY, YY). Different lowercase letters indicate significant differences between rhizosphere and bulk compartments ($p < 0.05$). Different uppercase letters indicate significant differences among the three sampling sites within the same soil compartment ($p < 0.05$). URE: urease; SUC: sucrase; CAT: catalase; S-NP: soluble neutral phosphatase. R: rhizosphere soil; BS: bulk soil. MZ: mengzhou; LY: Liangyuan; YY: yuanyang.

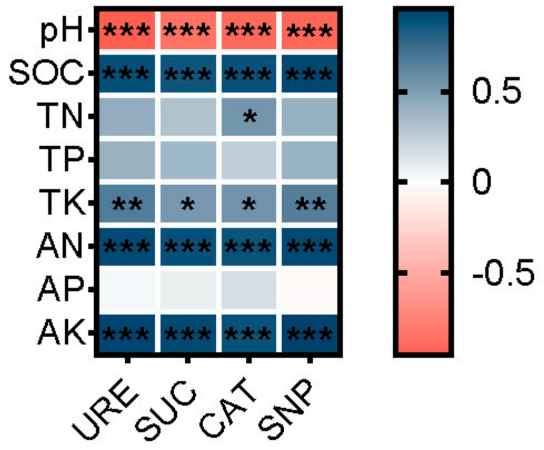

Figure S2. Heatmap of correlations of soil properties with enzyme activities. Blue and red colors represent positive and negative correlations, respectively; * $p < 0.05$, ** $p < 0.01$, *** $p < 0.001$.

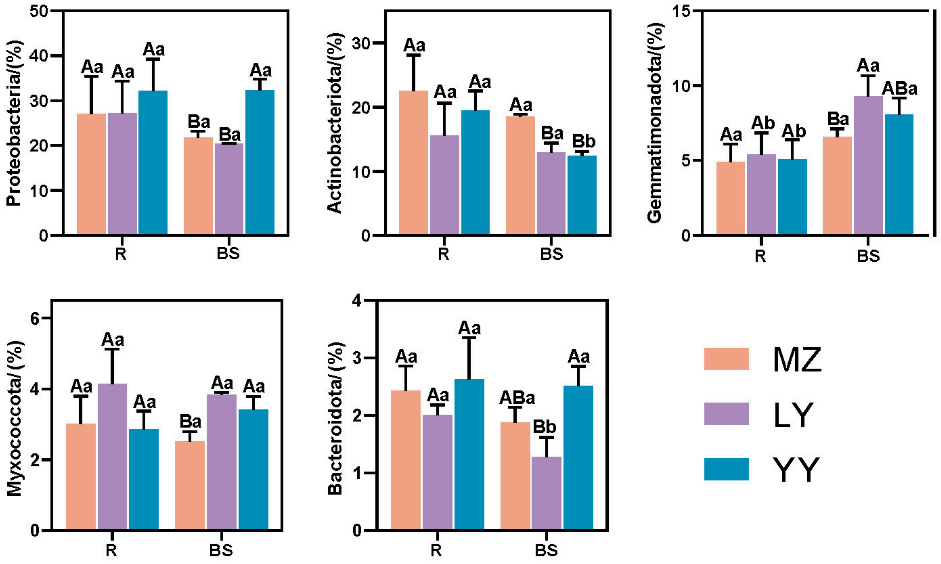

Figure S3. Relative abundance of bacterial phyla with significant variation across sampling sites and rhizosphere compartments. Lowercase letters indicate significant differences among sampling sites within the same rhizosphere compartment ($p < 0.05$). Uppercase letters indicate significant differences between rhizosphere and bulk compartments within the same sampling site ($p < 0.05$). R: rhizosphere soil; BS: bulk soil. MZ: mengzhou; LY: Liangyuan; YY: yuanyang.

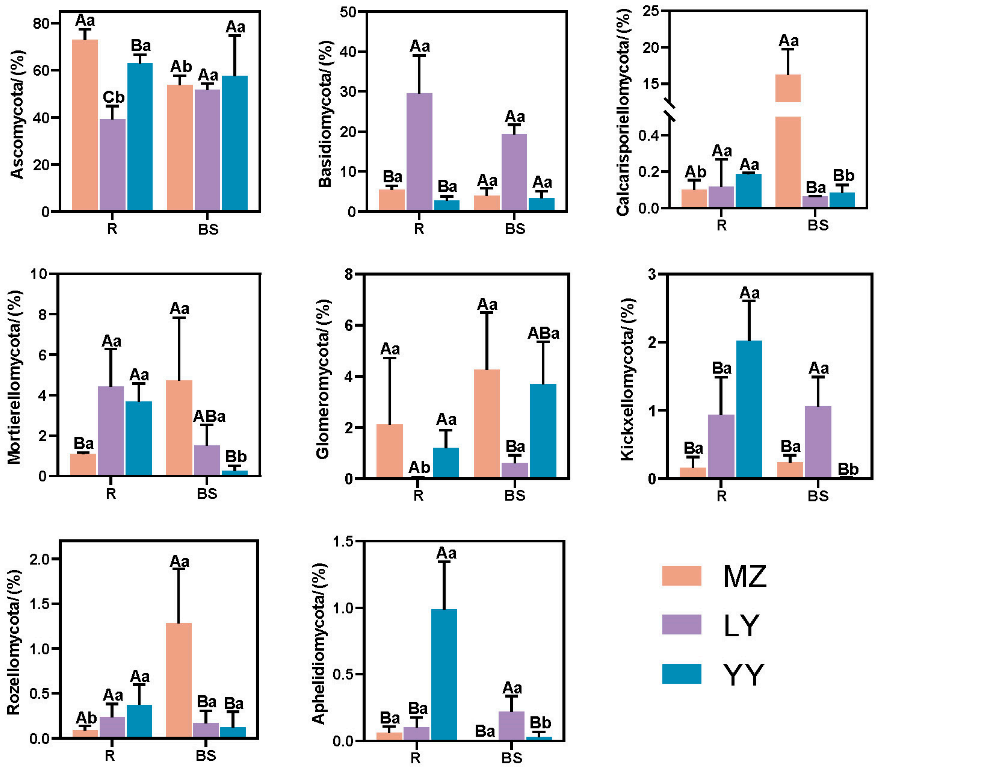

Figure S4. Relative abundance of fungal phyla with significant variation across sampling sites and two soil compartments. Lowercase letters indicate significant differences among sampling sites within the same rhizosphere compartment ($p < 0.05$). Uppercase letters indicate significant differences between rhizosphere and bulk compartments within the same sampling site ($p < 0.05$). R: rhizosphere soil; BS: bulk soil. MZ: mengzhou; LY: Liangyuan; YY: yuanyang.

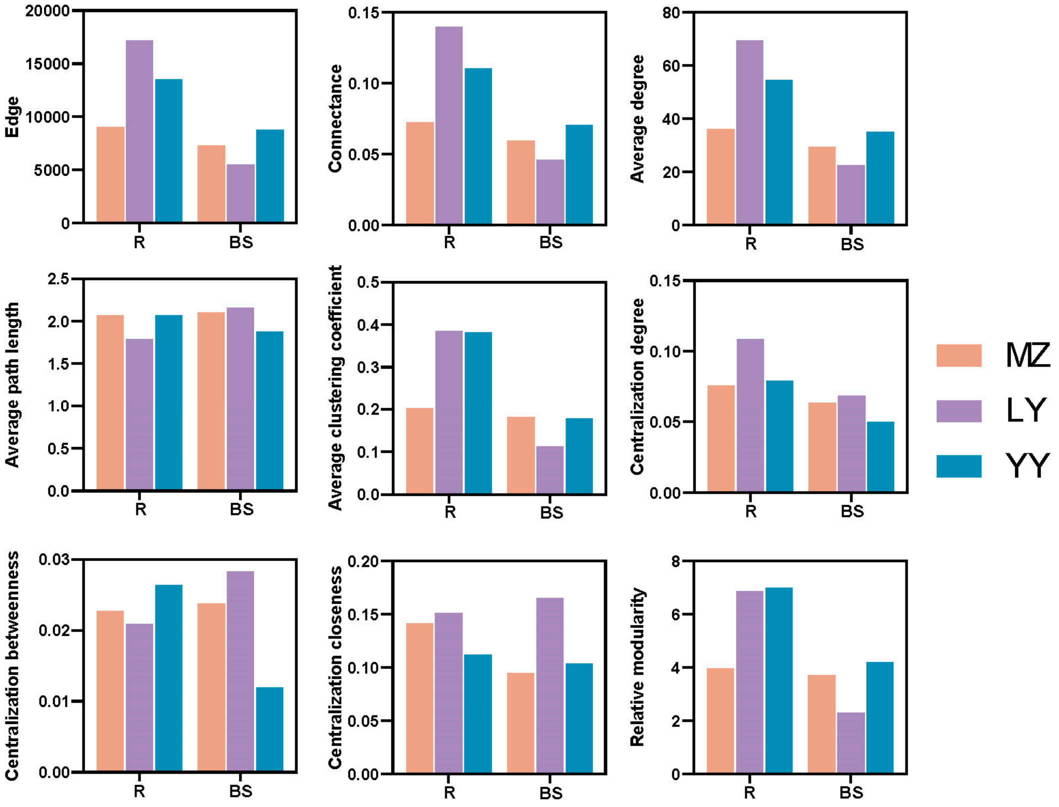

Figure S5. Microbial co-occurrence network topological properties differ between sampling sites and rhizosphere compartments. R: rhizosphere soil; BS: bulk soil. MZ: mengzhou; LY: Liangyuan; YY: yuanyang.

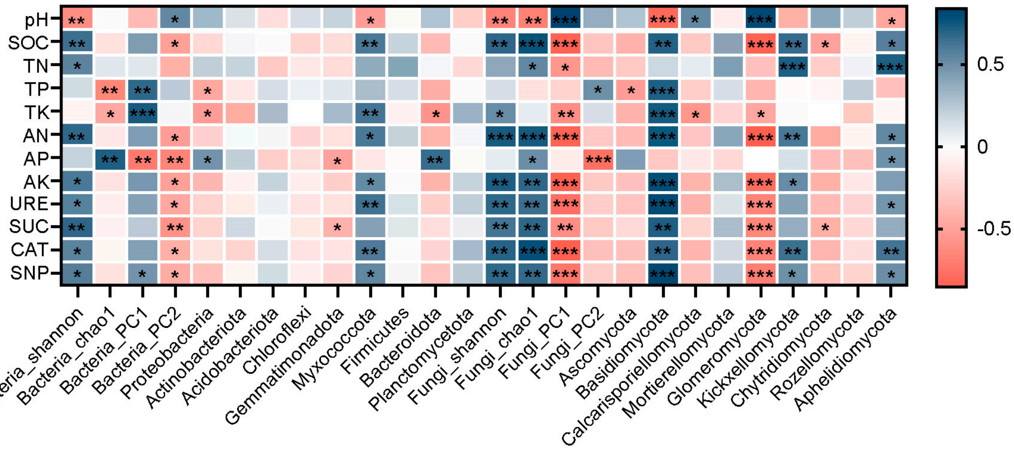

Figure S6. Heatmap of correlations of soil properties, enzyme activities with bacterial/fungal diversity and community composition. Blue and red colors represent positive and negative correlations, respectively; * $p < 0.05$, ** $p < 0.01$, *** $p < 0.001$.

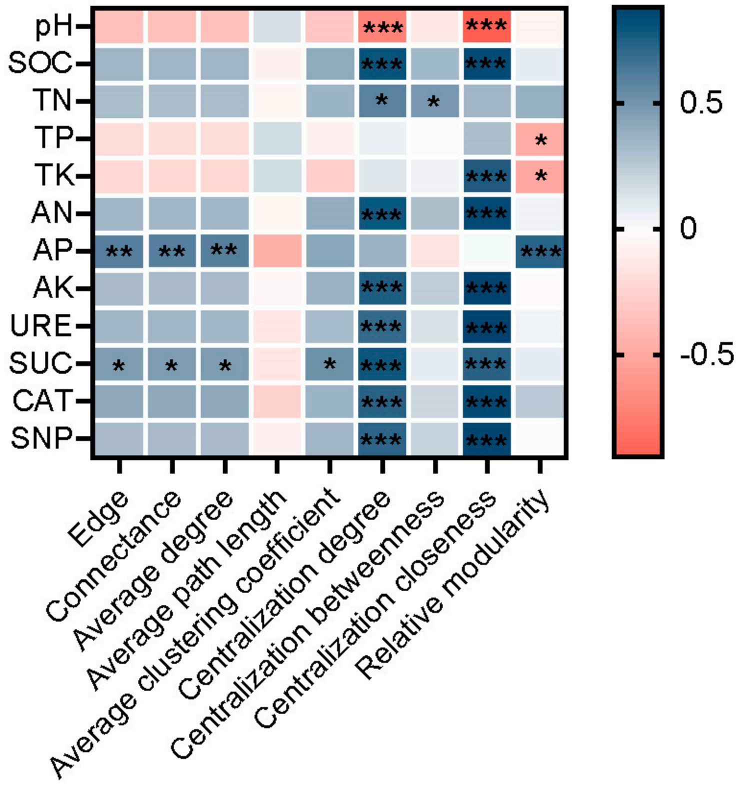

Figure S7. Heatmap of correlations of soil properties, enzyme activities with microbial co-occurrence network topological properties. Blue and red colors represent positive and negative correlations, respectively; * $p < 0.05$, ** $p < 0.01$, *** $p < 0.001$.

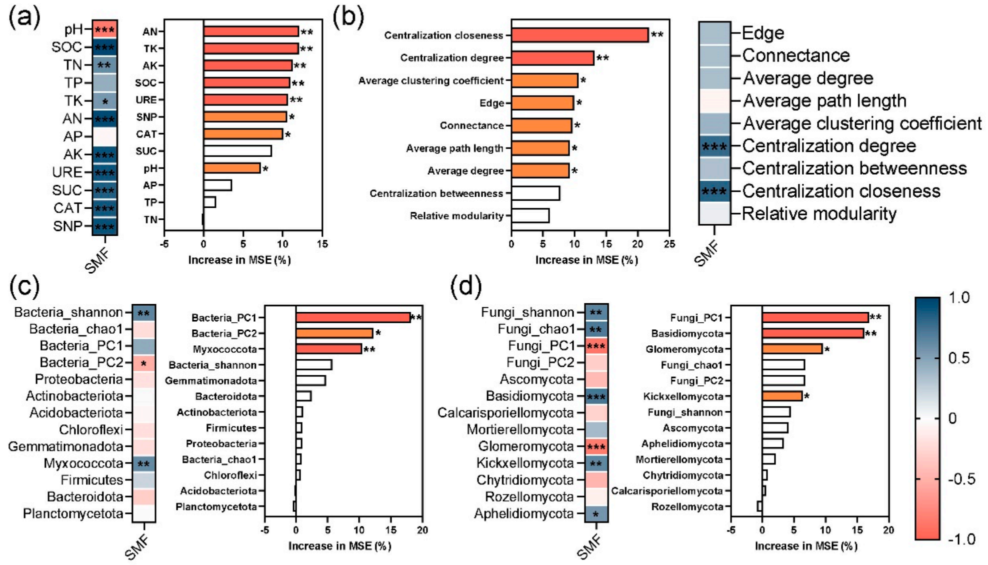

Figure S8. Correlation and random forest analysis of factors associated with soil multifunctionality. (a) Soil chemical properties and enzyme activities; (b) microbial network topological properties; (c) bacterial diversity and community composition; (d) fungal diversity and community composition. Blue and red colors represent positive and negative correlations, respectively; * $p < 0.05$, ** $p < 0.01$, *** $p < 0.001$.
